# 

**Published:** 1884-06

**Authors:** 


### CONTENTS:

Art. I.—The Wisconsin Dental College. By Adolph Peterman, D. D. S..............50
Art. II—Diseases of the Antrum. By Frank Abbott, M. D..........................53
Art. III—The First Permanent Molar and Its Early Management.
By J. Hardeman, D. D. S.......................................59
Art. IV.—Treatment of Pyorrhea Alveolaris. By A. W. Harlan, D. D. S............64
Art. V.—On the Disposition of Time and Its Relation to Fees.
By A. W. Harlan, D. D. S.......................................68
Art. VI.—Adapting Artificial Crowns to Roots of Teeth. By Geo. W. Smith. .....73
Art. VII.—The American Medical Association—Section on Oral and Dental Surgery 78
Art. VIII.—New Remedies. By Dr. Wm. A. Pease...................................81
Art. IX.—Influence of Micro-Organisms in the Production of Caries of the Teeth.
By A. S. Underwood, L. D. S., [Continetcued.]...................85
Meeting of Delegates.............................................87
National Association of Dental Examiners.........................88
American Dental Association......................................88
Pennsylvania State Dental Society,...............................88

### EDITORIAL, ETC.

Dental Education.................................................................89

### MONTHLY SUMMARY.

Dental Irritation Causing Spasms.................................................91
A New Remedy For Spasms..........................................................93
Ether............................................................................94
Excision of the Tongue...........................................................95
Effects of Tobacco Upon Boys.....................................................95
Trial Plates for the Lower Jaw...................................................96

